# Elevated Serum TSH Levels and TPOAb Positivity in Early Pregnancy are Associated with Increased Risk of Hypertensive Disorders of Pregnancy: A Prospective Cohort Study

**DOI:** 10.7150/ijms.103874

**Published:** 2025-01-01

**Authors:** Minhui Hu, Shen Gao, Kaikun Huang, Xueran Wang, Juan Li, Shuangying Li, Zhan Li, Wentao Yue, Shaofei Su, Enjie Zhang, Shuanghua Xie, Jianhui Liu, Yue Zhang, Yingyi Luan, Ruixia Liu, Chenghong Yin

**Affiliations:** Department of Central Laboratory, Beijing Obstetrics and Gynecology Hospital, Capital Medical University, Beijing Maternal and Child Health Care Hospital, Beijing 100026, China.

**Keywords:** Thyroid-stimulating hormone, Free thyroxine, Thyroid peroxidase antibody, Hypertensive disorders of pregnancy, Blood pressure

## Abstract

**Background:** The relationship between maternal thyroid-stimulating hormone (TSH), free thyroxine (FT4) and thyroid peroxidase antibody (TPOAb) status and hypertensive disorders of pregnancy (HDP) remains uncertain.

**Methods:** This was a prospective cohort study based on the China Birth Cohort Study (CBCS). 36,256 women were included at 6 to 13^+6^ gestation from February 2018 to December 2020. Generalized linear mixed models were used to investigate the association between thyroid function and HDP/BP. We further performed multiple subgroup analyses to test the robustness of this association.

**Results:** The final study population was 25,608, and the overall incidence of HDP was 8.0%. After adjusting for maternal age, pre-pregnancy BMI, education, household annual income, smoking status, conception method and parity, the odds of HDP increased by 3.0% with a 1-unit increase in TSH (OR 1.03, 95% CI 1.04-1.06). Maternal TSH and TPOAb positivity were associated with a higher risk of preeclampsia or eclampsia but not gestational hypertension (TSH: OR 1.04, 95% CI 1.01-1.07; TPOAb positivity: OR 1.30, 95% CI 1.09-1.56). TSH and TPOAb positivity were significantly and positively associated with systolic pressure (TSH: β 0.02, 95% CI 0.07-0.26; TPOAb positivity: β 0.02, 95% CI 0.12-0.98) and diastolic pressure (TSH: β 0.02, 95% CI 0.02-0.17; TPOAb positivity: β 0.02, 95% CI 0.06-0.75). Subgroup analyses suggested that the association between TSH and diastolic pressure was stronger in those with BMI ≥ 25 kg/m^2^ (*P* = 0.014).

**Conclusions:** Our founds suggest that high TSH and TPOAb positivity in the first trimester are associated with an increased risk of preeclampsia or eclampsia.

## Introduction

Hypertensive disorders of pregnancy (HDP) affect approximately 10% of all pregnancies, and are a primary contributor to both maternal and fetal morbidity and mortality^1^. Women with HDP are particularly susceptible to severe complications and even death. The long-term cardiovascular risk associated with HDP in women has been well-documented even decades after pregnancy^2^. Moreover, HDP may be linked to stillbirth and infant mortality and significantly increases the risk of adverse perinatal outcomes, such as preterm birth and offspring neurodevelopmental disorders^3^, ^4^. Notably, elevated maternal blood pressure (BP), even below the diagnostic criteria for HDP, has been reported to be associated with adverse pregnancy outcomes^5^, ^6^. Despite the relatively high incidence and severe complications related to HDP, its pathogenesis and risk factors remain not fully understood.

Thyroid receptors have been identified in both myocardial and vascular endothelial tissues, demonstrating sensitivity to fluctuations in circulating thyroid hormones^7^. Dyslipidemia and endothelial dysfunction caused by thyroid dysfunction and the direct effects of thyroid hormones on the myocardium can adversely affect the cardiovascular system^8^. The most frequently used biomarkers of thyroid function include thyroid-stimulating hormone (TSH), free thyroxine (FT4), and thyroid peroxidase antibody (TPOAb). In recent years, several studies have investigated the association between thyroid dysfunction and HDP, suggesting that higher TSH levels and/or lower FT4 levels were associated with a higher risk of HDP^9^, ^10^. However, other studies have shown inconsistent results^11^-^13^. Most of these studies did not account for TPOAb status, the presence of TPOAb positivity can weaken the response of the thyroid to HCG during pregnancy and affect the cut-off value of TSH. Ignoring TPOAb in studies may underestimate the true effect of TSH or FT4 on HDP during pregnancy^14^. Additionally, thyroid function is altered during pregnancy, and the late gestational age of thyroid function testing may be one reason for the inconsistent results of previous studies. Moreover, there is a scarcity of prospective studies that examine the correlation between TSH and FT4, considered as continuous variables, and HDP, which may overlook the effect of mild changes in thyroid function indicators on HDP.

High TSH levels have been reported to be associated with increased systolic pressure (SBP) and/or diastolic pressure (DBP) in non-pregnant populations^15^, ^16^. Due to pregnancy-specific physiology, TSH, FT4, and TOPAb concentrations differ from those in the non-pregnant state. Only one small cohort study has explored the association between thyroid dysfunction during pregnancy and maternal BP, and it did not include TPOAb status data^17^.

Evidence on the relationship between maternal thyroid function and HDP is limited. The 2017 ATA guidelines have yet to definitively establish the correlation between TSH, FT4, and TPOAb with HDP. Consequently, additional prospective research is warranted to elucidate the correlation between elevated TSH, FT4, TPOAb, and HDP. We conducted a population-based cohort study to assess the relationship between maternal thyroid function biomarkers (TSH, FT4, TPOAb positivity) at 6-13^+6^ weeks of gestation and the risk of HDP, based on the China Birth Cohort Study (CBCS).

## Methods

### Study population

This prospective cohort study was based on the CBCS, which has been described elsewhere^18^. Participants were recruited from the Beijing Obstetrics and Gynecology Hospital, Capital Medical University, between February 2018 and December 2020. 36,256 pregnant women were initially enrolled at 6-13^+6^ weeks of gestation, the exclusion criteria included: (a) request to withdraw from the study; (b) ultrasound-confirmed non-singleton pregnancy in the first trimester; (c) a history of thyroid disease (i.e. pre-pregnancy thyroid dysfunction, thyroid surgery, thyroiditis, thyroid cysts ,or thyroid tumor); (d) pre-existing hypertension or systolic BP ≥140 mmHg and/or diastolic BP ≥90 mmHg in the first trimester; (e) use of medications that affect thyroid function, or blood pressure prior to pregnancy (i.e. L-T4, labetalol, propylthiouracil, prednisone, methylprednisolone, dexamethasone, or budesonide); (f) Missed data for thyroid function; (g) Abortion or induction of labor; and (h) no discharge diagnosis. The final study population consisted of 25,608 pregnant women. According to previous studies, the odds ratio (OR) between thyroid function and HDP was 1.20-1.53^19^-^21^, while the prevalence of HDP in our population was 8%. Assuming an alpha (probability of type I error) of 0.05 and a delta (acceptable error) of 0.10, the estimated sample size was 1315-7157. Therefore, the actual sample size could be of sufficient statistical power.

This study was approved by the Ethics Committee of Beijing Obstetrics and Gynecology Hospital, Capital Medical University (reference number: 2018-KY-003-02). All subjects signed appropriate informed consent documents.

### Data collection

All pregnant women completed a standardized questionnaire during their initial antenatal visit that took place at 6-13^+6^ weeks of gestation. Maternal demographic and obstetric characteristics, including maternal age (<35 years/ ≥35 years), pre-pregnancy BMI (<25 kg/m^2^/ ≥25 kg/m^2^), ethnic (han/minority), maternal education (<16 years/≥16 years), household annual income (<200,000 CNY/≥200,000 CNY), smoking status (yes/no), alcohol consumption (yes/no), conception method (natural conception / Assisted Reproductive Technology (ART)), parity (nullipara/multipara). ART includes in vitro fertilization, intracytoplasmic sperm injection, embryo transfer. All of the above data were routinely collected and updated by the Electronic Data Capture System.

Maternal blood samples were collected at 6-13^+6^ weeks of gestation after an 8-12 h overnight fast. The serum concentrations of TSH, FT4 and TPOAb were measured via electrochemiluminescence immunoassay (ADVIA Centaur XP, Siemens Healthcare Diagnostics, Tarrytown, NY, USA) in the accredited clinical laboratory of the hospital. According to the kit instructions, TPOAb > 60 IU/L was defined as TPOAb positivity.

### Definition of maternal BP and HDP

Maternal BP measurements were assessed during routine prenatal visits (at 6-13^+6^ weeks' gestation, 20-24^+6^ weeks' gestation, and 28-34^+6^ weeks' gestation). Hypertension was diagnosed based on the mean of at least two measurements of SBP being ≥ 140 mmHg and/or DBP being ≥ 90 mmHg^22^.

HDP was defined according to international standards. Briefly, HDP included gestational hypertension and preeclampsia/eclampsia in this study. New onset of hypertension at ≥20 weeks of gestation was considered gestational hypertension. Preeclampsia was defined as gestational hypertension accompanied by proteinuria (24 h urine collection containing 300 mg or more of protein, or urine dipstick protein of 1^+^), or other maternal end-organ dysfunction (neurologic or visual dysfunction, pulmonary edema, hematological complications, acute kidney injury (AKI), liver involvement), or uteroplacental dysfunction. Eclampsia was defined as new-onset convulsions based on pre-eclampsia that cannot be explained by other causes^22^.

### Statistical analysis

Normality and homogeneity were assessed by Shapiro-Wilk and Bartlett's test for continuous variables. Continuous variables were expressed as mean ± standard deviation (SD) and categorical variables were expressed as frequency with percentage. Student's t-test or the chi-squared test was utilized to discern differences between the HDP group and the non-HDP group.

Generalized linear mixed models were used to investigate the association between thyroid function biomarkers (TSH, FT4, TPOAb positivity) and HDP/BP. Two models were constructed for confounders in this study. The crude model did not account for any potential confounders. Adjusted models were adjusted for maternal age, pre-pregnancy BMI, education, household annual income, smoking status, conception method and parity. Furthermore, to explore the possibility of a linear relationship between TSH and HDP, we transformed the continuous variables into categorical variables using quartiles, and the *P*-values for trend were calculated.

To verify the stability of the association of maternal thyroid function biomarkers (TSH and TPOAb positivity) with HDP and BP in different subgroups, stratified analyses were conducted by maternal age (<35 years/≥35 years), pre-pregnancy BMI (<25 kg/m^2^/ ≥25 kg/m^2^), maternal education (<16 years/≥16 years), household annual income (<200,000 CNY/ ≥200,000 CNY), conception method (natural conception/ART), and parity (nullipara/multipara)^23^. The calculation of *P*-values for group differences was executed.

Every statistical test was two-sided, and *P*-value < 0.05 was considered to indicate statistically significant results. All analyses were performed using SPSS 26.0 (SPSS, Inc., Chicago, IL, USA) and R version 4.1.1 (http://www.R-project.org).

## Results

### Baseline characteristics

**Figure 1:**
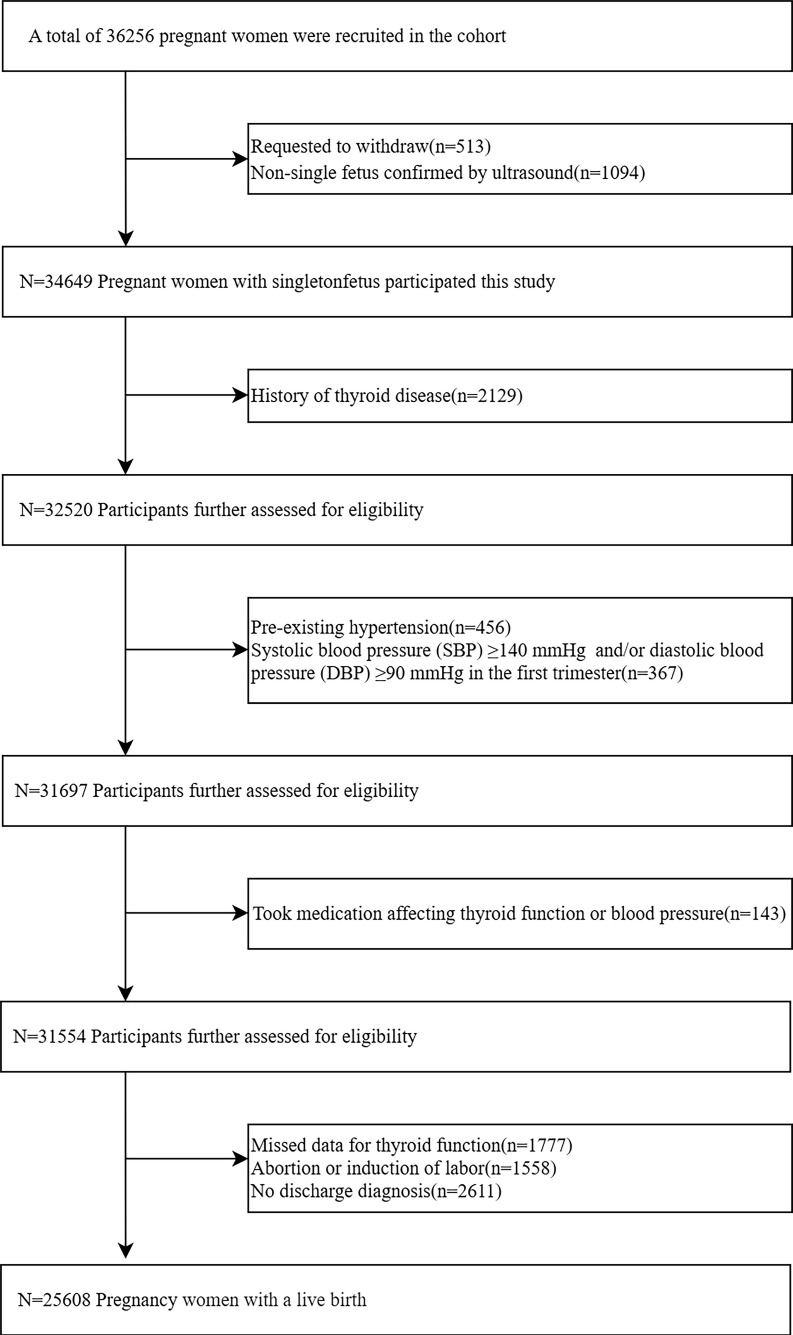
Flow chart of study participants selection.

The final study population consisted of 25,608 pregnant women with a live birth following the application of the exclusion criteria (**Figure 1**). In our study, 11.3% of the participants tested positive for TPOAb. A total of 2,059 participants (8.0%) were diagnosed with HDP. When compared with non-HDP participants, HDP participants had a higher proportion of age ≥35 years (30.5% versus 23.4%, *P* < 0.001) and pre-pregnancy BMI ≥25 kg/m^2^ (29.6% versus 11.1%, *P* < 0.001). Furthermore, compared to the non-HDP, the HDP group was more likely to have received < 16 years of education (82.6% versus 76.7%, *P* < 0.001), to earn less than 200,000 CNY (43.5% versus 37.1%, *P* < 0.001), to be a past smoker (3.9% versus 2.9%, *P* = 0.012), to have a greater incidence of ART (10.0% versus 5.3%, *P* < 0.001), and to be nullipara (57.7% versus 53.8%, *P* < 0.001). However, no significant disparities were noted in ethnicity, or alcohol consumption between the two groups (*P*=0.923; *P*=0.231, respectively) **(Table 1)**.

### Association between thyroid function and HDP

As shown in **Table 2**. When maternal TSH and FT4 were analyzed as continuous variables, TSH was found to significantly and positively correlate with HDP (OR 1.04, 95% CI 1.04-1.07). After adjusting for maternal age, pre-pregnancy BMI, education, household annual income, smoking status, pregnancy mode and parity, the risk of HDP increased by 3.0% with each 1-unit increase in TSH (OR 1.03, 95% CI 1.04-1.06). TSH was associated with a higher risk of preeclampsia or eclampsia but not gestational hypertension, after adjusting for confounding variables (OR 1.04, 95% CI 1.01-1.07). In comparison to TPOAb negativity, TPOAb positivity was associated with HDP only in the unadjusted model (OR 0.86, 95% CI 0.75-0.99). However, TPOAb positivity was associated with a higher risk of preeclampsia or eclampsia (OR 1.30, 95% CI 1.09-1.56) in the unadjusted model, and this association persisted after adjusting for maternal age, pre-pregnancy BMI, education, household annual income, smoking status, pregnancy mode and parity (OR 1.29, 95% CI 1.08-1.55). No significant correlation was identified between FT4 and the risk of HDP in either the unadjusted model or the adjusted model (OR 0.99, 95% CI 0.97-1.01; OR 1.01, 95% CI 0.99-1.03, respectively). FT4 was not found to be associated with gestational hypertension and preeclampsia or eclampsia after adjusting for maternal age, pre-pregnancy BMI, education, household annual income, smoking status, pregnancy mode and parity (OR 1.02, 95% CI 1.00-1.05; OR 0.99, 95% CI 0.96-1.02, respectively).

When maternal TSH was transformed into quartiles, the results indicated that compared to the lowest quartile (reference), the highest quartile (Q4) of TSH significantly increased the risk of HDP after adjusting for maternal age, pre-pregnancy BMI, education, household annual income, smoking status, pregnancy mode and parity (OR 1.23, 95% CI 1.08-1.40). And the odds of HDP increased significantly with each quartile increase in TSH (*P* for trend =0.001). Compared to the lowest quartile (reference), the highest quartile (Q4) of TSH significantly increased risk of preeclampsia or eclampsia after adjusting for all confounding factors (OR 1.24, 95% CI 1.04-1.48) and the trend test showed a statistically significant upward trend (*P* for trend =0.008) (**Table 3**). However, there were no discernible differences in the incidence rate of HDP between the FT4 quintile groups (OR_Q2_ 0.95, 95% CI 0.84-1.08; OR_Q3_ 0.98, 95% CI 0.86-1.11; OR_Q4_ 1.02, 95% CI 0.89-1.16; *P* for trend = 0.764).

### Association between thyroid function and BP

As shown in **Table 4,** unadjusted analysis indicated that TSH and TPOAb positivity in the first trimester were positively associated with SBP (TSH: standardized coefficients (β) 0.03, 95% CI 0.13-0.32; TPOAb positivity: β 0.02, 95% CI 0.16-1.04) and DBP (TSH: β 0.02, 95% CI 0.06-0.21; TPOAb positivity: β 0.02; 95% CI 0.09-0.79) in the third trimester. After adjusting for maternal age, pre-pregnancy BMI, education, household annual income, smoking status, pregnancy mode and parity, TSH and TPOAb positivity remained positively associated with SBP (TSH: β 0.02, 95% CI 0.07-0.26; TPOAb positivity: β 0.02; 95% CI 0.12-0.98) and DBP (TSH: β 0.02, 95% CI 0.02-0.17; TPOAb positivity: β 0.02, 95% CI 0.06-0.75). No significant correlation was identified between FT4 and BP in the adjusted model (SBP: β 0.00, 95% CI -0.05-0.06; DBP: β 0.01; 95% CI 0.00-0.08).

### Subgroup analysis

Subgroup analyses were carried out based on maternal age, pre-pregnancy BMI, maternal education, household annual income, conception method, and parity. The results demonstrated stronger associations between TSH levels and SBP among individuals with natural conception than those with ART (*P* = 0.014). Additionally, pre-pregnancy BMI significantly modified the association between TSH levels and DBP. Specifically, the association between TSH levels and the DBP was stronger among individuals with a pre-pregnancy BMI ≥ 25 kg/m^2^ compared to those with a pre-pregnancy BMI<25 kg/m^2^ (*P* = 0.014). No other factors were found to significantly affect the correlations between maternal thyroid function (TSH and TPOAb positivity) and the incidence of preeclampsia or eclampsia/BP (**Figure S1-6**).

## Discussion

This population-based prospective cohort study determined that the risk of subsequent HDP was positively correlated with TSH levels and TPOAb positivity during early pregnancy. Elevated TSH levels and TPOAb positivity were associated with an increased risk of preeclampsia or eclampsia, but not gestational hypertension. Furthermore, maternal TSH levels and TPOAb positivity in the first trimester were positively associated with SBP and DBP in the third trimester. The results of this study indicate that elevated TSH levels and the presence of TPOAb in early pregnancy could potentially act as risk factors for the development of HDP.

The analysis of over 25,000 pregnant women revealed a correlation between the TSH levels during early pregnancy and the likelihood of developing HDP. A study conducted by Karen *et al.*, including 24,883 pregnant women, showed that the risk of HDP increased with rising serum TSH levels within the general population (*P* for trend =0.004)^9^. Our results are broadly consistent with theirs, although their results were not adjusted for BMI. A retrospective study by Manisto *et al.* examined the relationship between hypothyroidism or hyperthyroidism and HDP in a cohort of 223,512 US participants^24^. The results indicated that both hypothyroidism and hyperthyroidism significantly increased the risk of preeclampsia (OR 1.47, 95% CI 1.20-1.81; OR 1.78, 95% CI 1.08-2.94, respectively). Our study reveals that elevated TSH levels are associated with a significantly increased risk of HDP, mainly due to an elevated risk of preeclampsia or eclampsia rather than gestational hypertension. It has been found that the development of preeclampsia may be associated with abnormal expression patterns of macroautophagy and increased placental calcifications, and whether this change is associated with alterations in thyroid function needs further study^25^, ^26^. A preliminary observational study conducted on pregnant women diagnosed with HDP showed a significant positive relationship between TSH levels and systemic vascular resistance (β 0.162, 95% CI 0.009-0.308) and a significant negative relationship between TSH levels and cardiac output (β -0.260, 95% CI -0.392 - -0.103)^27^. These findings suggest that elevated TSH levels may be a risk factor affecting maternal hemodynamics. We also observed that maternal TSH levels in the first trimester were positively associated with SBP and DBP in the third trimester. These results align with prior studies conducted in diverse populations^15^, ^16^. Additionally, in subgroup analysis, we found that pregnant women with pre-pregnancy BMI ≥ 25 kg/m^2^ were more likely to have elevated DBP in the third trimester. This may be related to the fact that leptin, secreted by adipose tissue, can promote TSH secretion^28^, ^29^. Based on these results, obstetricians and endocrinologists should be alert to the potential harms of high TSH levels in the first trimester for pregnant women, especially those with pre-pregnancy BMI ≥ 25 kg/m^2^. Pregnant women with TSH levels ≥ 2.04 mU/L or TPOAb positivity in the first trimester are encouraged to measure home BP regularly and remain vigilant about any fluctuations in their BP throughout the pregnancy. Furthermore, managing a pre-pregnancy BMI < 25 kg/m^2^ could potentially mitigate the risk of developing HDP. For those pregnant women who have already been diagnosed with HDP, closely monitoring TSH levels during the HDP treatment might serve as a preventive measure against its further progression.

In this study cohort, 11.3% of pregnant women tested positive for TPOAb, consistent with findings from previous studies on pregnant women in China^30^. TPOAb is the most prevalent anti-thyroid autoantibody^31^. The 2017 ATA guidelines advocate for the evaluation of TPOAb in testing for thyroid autoimmunity^32^. The finding in the present study that TPOAb positivity in the first trimester was associated with an increased risk of preeclampsia or eclampsia was supported by similar results from a case-control study^33^. Moreover, this study is the first to demonstrate a positive association between TPOAb positivity in the first trimester and elevated SBP and DBP in the third trimester. However, the underlying mechanism between TPOAb positivity and HDP remains hypothetical. Evidence suggests that thyroid antibodies disrupt the balance between Th1/Th2/Th17 and T regulatory systems, leading to immune dysregulation^34^, ^35^. Abnormal expression of Th17 cells is associated with pregnancy-related diseases such as preeclampsia^36^. TPOAb positivity has also been reported to be associated with impaired thyroid response to human chorionic gonadotropin during pregnancy and is a major risk factor for thyroid dysfunction 14. Besides, the presence of TPOAb positivity could potentially indicate an underlying thyroid insufficiency, characterized by a diminished thyroid capacity to adapt to physiological alterations during pregnancy^37^.

Contrary to the findings presented above, several other studies failed to identify any correlation between TSH or TPOAb positivity and HDP^11^-^13^. Several factors may contribute to the inconsistencies observed in current studies, including the assessment of thyroid function at varying gestational ages, ethnic and geographic disparities, and failure to control for known confounding factors associated with thyroid parameters and/or HDP risk.

The mechanism by which TSH contributes to HDP has not been elucidated, with several potential explanations: First, through its affinity for the TSH receptor, elevated serum TSH can induce vascular endothelial dysfunction. Previous studies have demonstrated that TSH induces endothelial nitric oxide synthase (eNOS) uncoupling, which not only leads to a decrease in endothelial NO physiological activity, a key indicator of vascular endothelial dysfunction, but also leads to an increase in superoxide anion concentration, which exacerbates oxidative stress^38^. Additionally, it can affect the proliferation and permeability of endothelial cells and promote abnormal proliferation and apoptosis^39^. Second, TSH, possibly acting as a neurotransmitter, appears to be associated with cardiac autonomic dysfunction and an imbalance in sympathetic nervous system activity^40^, ^41^. Third, patients with elevated TSH are at increased risk of cardiovascular events due to both hypercoagulable states and dyslipidemia^42^.

The current study possesses several notable strengths. First, the early gestational age of thyroid function testing (6-13^+6^ weeks) greatly reduces the variation of thyroid function due to gestational age. Furthermore, to our knowledge, this study is the first to confirm the association between maternal TPOAb and BP during pregnancy. It is also the inaugural prospective study to examine the relationship between TSH and FT4 as continuous variables with HDP. However, several limitations to this study should be acknowledged. To begin, we did not have the data on daily iodine intake and urinary iodine excretion to accurately assess the participants' iodine status during early pregnancy. Recent research has shown that iodine consumption significantly influences normal serum TSH levels^43^. However, China has adopted a universal salt iodization policy, and a previous study evaluated the iodine nutritional status of pregnant women in China using thyroid function and urinary iodine concentration and concluded that the iodine status of these women was generally adequate. As a result, we may safely assume that this is a study in an iodine-sufficient area^44^ In addition, this is a prospective single-center cohort study conducted at a tertiary maternal and child health care hospital in Beijing, the capital of China, where the nutritional status of pregnant women was adequate. Next, our study was a single-center study, and more than 90% of the participants were of the Han nationality, potentially restricting the generalizability of the results to diverse ethnic or regional populations. Additionally, our study was limited by the availability of thyroid function data only for the first trimester. Consequently, future research with larger sample sizes that encompass both the second and third trimesters is imperative to provide a holistic understanding of the association throughout pregnancy.

## Conclusion

The results of this study indicated a significant correlation between TSH levels and TPOAb status during early pregnancy and HDP. In addition, this study also found that TSH level and TPOAb positivity in the first trimester were positively correlated with BP in the third trimester. Elevated TSH level and TPOAb positivity may be risk factors for HDP. This study emphasized that early detection of maternal TSH levels and TPOAb status may be one of the important measures to prevent the development of HDP.

## Supplementary Material

Supplementary figures.

## Figures and Tables

**Table 1:** General characteristics between the HDP group and the non-HDP group

Characteristics	Non-HDP (n=23,549)	HDP (n=2059)	Gestational hypertension (n=1015)	Preeclampsia or eclampsia (n=1044)	*^a^ P-*value
Maternal age, n (%)					**<0.001**
<35 years,	18,041 (76.60)	1432 (69.5)	699 (68.9)	733 (70.2)	
≥35 years,	5508 (23.4)	627 (30.5)	316 (31.1)	311 (29.8)	
Pre-pregnancy BMI, n (%)					**<0.001**
<25 kg/m^2^,	20,933 (88.9)	1450 (70.4)	715 (70.4)	735 (70.4)	
≥25 kg/m^2^,	2616 (11.1)	609 (29.6)	300 (29.6)	309 (29.6)	
Ethnic, n (%)					0.923
Han	21,728 (92.3)	1901 (92.3)	942 (92.8)	959 (91.9)	
Minority	1821 (7.7)	158 (7.7)	73 (7.2)	85 (8.1)	
Maternal education, n (%)					**<0.001**
<16 years	18,053 (76.7)	1701 (82.6)	832 (82.0)	869 (83.2)	
≥16 years	5496 (23.3)	358 (17.4)	183 (18.0)	175 (16.8)	
Household annual income, n (%)					**<0.001**
<200,000, CNY	8725 (37.1)	896 (43.5)	435 (42.9)	462 (44.3)	
≥200,000, CNY	14,824 (62.9)	1163 (56.5)	580 (57.1)	582 (55.7)	
Smoking status, n (%)					**0.012**
Yes	684 (2.9)	80 (3.9)	978 (96.4)	1001 (95.9)	
No	22,865 (97.1)	1979 (96.1)	37 (3.6)	43 (4.1)	
Alcohol consumption, n (%)					0.231
Yes	926 (3.9)	70 (3.4)	33 (3.3)	37 (3.5)	
No	22,623 (96.1)	1989 (96.6)	982 (96.7)	1007 (96.5)	
Conception method, n (%)					**<0.001**
Natural conception	22,312 (94.7)	1855 (90.0)	918 (90.4)	937 (89.8)	
ART	1237 (5.3)	204 (10.0)	97 (9.6)	107 (10.2)	
Parity, n (%)					**<0.001**
Nullipara	12,673 (53.8)	1189 (57.7)	568 (56.0)	621 (59.5)	
Multipara	10,876 (46.2)	870 (42.3)	447 (44.0)	423 (40.5)	
Thyroid function parameters					
TSH, mU/L, mean ± SD	1.52 ± 1.36	1.62 ± 1.26	1.59 ± 1.23	1.65 ± 1.29	**0.001**
FT4, pmol/L, mean ± SD	16.10 ± 2.43	16.32 ± 2.58	16.40 ± 2.49	16.23 ± 2.66	0.353
TPOAb positivity, n (%)	2897 (11.3)	273 (12.8)	115 (11.3)	147 (14.1)	**0.035**

^a^ Differences in these variables between the non-HDP group and HDP group were calculated using the student's t-test or chi-square test, as appropriate. *P* < 0.05 was considered to be statistically significant. Abbreviation: SD, standard deviation; HDP, hypertensive disorders of pregnancy; BMI, body mass index. ART, Assisted Reproductive Technology. ART includes in vitro fertilization, intracytoplasmic sperm injection, embryo transfer; TSH, thyroid-stimulating hormone; FT4, free thyroxine; TPOAb, thyroid peroxidase antibody.

**Table 2:** OR estimates for the association between thyroid function and HDP, gestational hypertension, preeclampsia or eclampsia

	Crude Model OR (95%CI)	Adjusted Model OR (95%CI)
HDP		
TSH	**1.04 (1.04,1.07)**	**1.03 (1.04,1.06)**
FT4	0.99 (0.97,1.01)	1.01 (0.99,1.03)
TPOAb positive	**0.86 (0.75,0.99)**	1.15 (1.00,1.32)
Gestational hypertension		
TSH	1.03 (0.99,1.06)	1.02 (0.98,1.06)
FT4	1.01 (0.98,1.03)	1.02 (1.00,1.05)
TPOAb positive	1.00 (0.82,1.22)	0.99 (0.81,1.21)
Preeclampsia or eclampsia		
TSH	**1.05 (1.01,1.08)**	**1.04 (1.01,1.07)**
FT4	0.98 (0.95,1.00)	0.99 (0.96,1.02)
TPOAb positive	1.30 (1.09,1.56)	**1.29 (1.08,1.55)**

Crude Model: Unadjusted model. Adjusted Model: adjusted for maternal age, pre-pregnancy BMI, education, household annual income, smoking status, conception method and parity. Abbreviation: HDP, hypertensive disorders of pregnancy; TSH, thyroid-stimulating hormone; FT4, free thyroxine; TPOAb, thyroid peroxidase antibody.

**Table 3:** Risk of HDP, gestational hypertension, preeclampsia or eclampsia at different levels of TSH and FT4

	Q1	Q2	Q3	Q4	*P* for trend
HDP					
TSH	reference	1.06 (0.93,1.21)	1.09 (0.96,1.25)	**1.23 (1.08,1.40)**	**0.001**
FT4	reference	0.95 (0.84,1.08)	0.98 (0.86,1.11)	1.02 (0.89,1.16)	0.764
Gestational hypertension					
TSH	reference	1.10 (0.92,1.33)	1.12 (0.93,1.35)	1.19 (0.99,1.43)	0.069
FT4	reference	0.96 (0.80,1.15)	1.08 (0.90,1.29)	1.11 (0.93,1.33)	0.163
Preeclampsia or eclampsia					
TSH	reference	1.01 (0.84,1.22)	1.06 (0.88,1.27)	**1.24 (1.04,1.48)**	**0.008**
FT4	reference	0.95 (0.80,1.13)	0.89 (0.75,1.06)	0.93 (0.78,1.11)	0.333

The models were adjusted for maternal age, pre-pregnancy BMI, education, household annual income, smoking status, conception method and parity. Abbreviation: OR, odds ratio; Q, quantile; HDP, hypertensive disorders of pregnancy. TSH, thyroid-stimulating hormone; FT4, free thyroxine. Q1 of TSH was ≤ 0.72 mU/L, Q2 of TSH was 0.73-1.28 mU/L, Q3 of TSH was 1.29-2.03 mU/L, and Q4 of TSH was ≥2.04 mU/L. Q1 of FT4 was ≤ 14.88 pmol/L, Q2 of FT4 was 14.89-16.14 pmol/L, Q3 of FT4 was 16.15-17.57 pmol/L, and Q4 of FT4 was ≥17.58 pmol/L.

**Table 4:** Association between thyroid function and maternal blood pressure in the third trimester.

	Crude Model β (95% CI)	Adjusted Model β (95% CI)
SBP		
TSH	**0.03 (0.13,0.32)**	**0.02 (0.07,0.26)**
FT4	-0.01 (-0.10,0.01)	0.00 (-0.05,0.06)
TPOAb positivity	**0.02 (0.16,1.04)**	**0.02 (0.12,0.98)**
DBP		
TSH	**0.02 (0.06,0.21)**	**0.02 (0.02,0.17)**
FT4	0.00 (-0.03,0.05)	0.01 (0.00,0.08)
TPOAb positivity	**0.02 (0.09,0.79)**	**0.02 (0.06,0.75)**

P < 0.05 was considered to be statistically significant. Crude Model: Unadjusted model. Adjusted Model: adjusted for maternal age, pre-pregnancy BMI, education, household annual income, smoking status, conception method and parity. Abbreviation: BP, blood pressure, SBP, systolic blood pressure; DBP, diastolic blood pressure; TSH, thyroid-stimulating hormone; FT4, free thyroxine; TPOAb, thyroid peroxidase antibody.

## Abbreviations

BMI: body mass index
CBCS: China Birth Cohort Study
BP: blood pressure
HDP: hypertensive disorders of pregnancy
FT4: free thyroxine
TPOAb: thyroid peroxidase antibody
TSH: thyroid-stimulating hormone
ART: Assisted Reproductive Technology
