# Supplementary material for: Elevated Serum TSH Levels and TPOAb Positivity in Early Pregnancy are Associated with Increased Risk of Hypertensive Disorders of Pregnancy: A Prospective Cohort Study
Source: Int J Med Sci. 2025 Jan 1;22(3):575–84. doi: 10.7150/ijms.103874 (PMC11783071; doi:10.7150/ijms.103874)
Supplement: Supplementary file 1 — Supplementary figures. [file ijmsv22p0575s1.pdf]

Supplementary Materials

Elevated Serum TSH Levels and TPOAb Positivity in Early Pregnancy are Associated with Increased Risk of Hypertensive Disorders of Pregnancy: A Prospective Cohort Study

Minhui Hu,[1,†] Shen Gao,[1,†] Kaikun Huang,[1] Xueran Wang,[1] Juan Li,[1] Shuangying Li,[1] Zhan Li,[1] Wentao Yue,[1] Shaofei Su,[1] Enjie Zhang,[1] Shuanghua Xie,[1] Jianhui Liu,[1] Yue Zhang,[1] Yingyi Luan,[1*] Ruixia Liu,[1*] Chenghong Yin.[1*]

[1] Department of Central Laboratory, Beijing Obstetrics and Gynecology Hospital, Capital Medical University, Beijing Maternal and Child Health Care Hospital, Beijing 100026, China

[†] These authors share first authorship.

* Corresponding author

Yingyi Luan

Email: luanyingyi@mail.ccmu.edu.cn

ORCID: 0000-0002-6986-1701

Tel.: +86-10-52277397. Address: No. 251 Yaojiayuan Road, Chaoyang District, Beijing 100026, China

Ruixia Liu

Email: liuruixia@ccmu.edu.cn

ORCID: 0000-0001-5835-4424

Tel.: +86-10-52277607. Address: No. 251 Yaojiayuan Road, Chaoyang District, Beijing 100026, China

Chenghong Yin

Email: yinchh@ccmu.edu.cn

ORCID: 0000-0002-2503-3285

Tel.: +86-10-85968401. Address: No. 251 Yaojiayuan Road, Chaoyang District, Beijing 100026, China

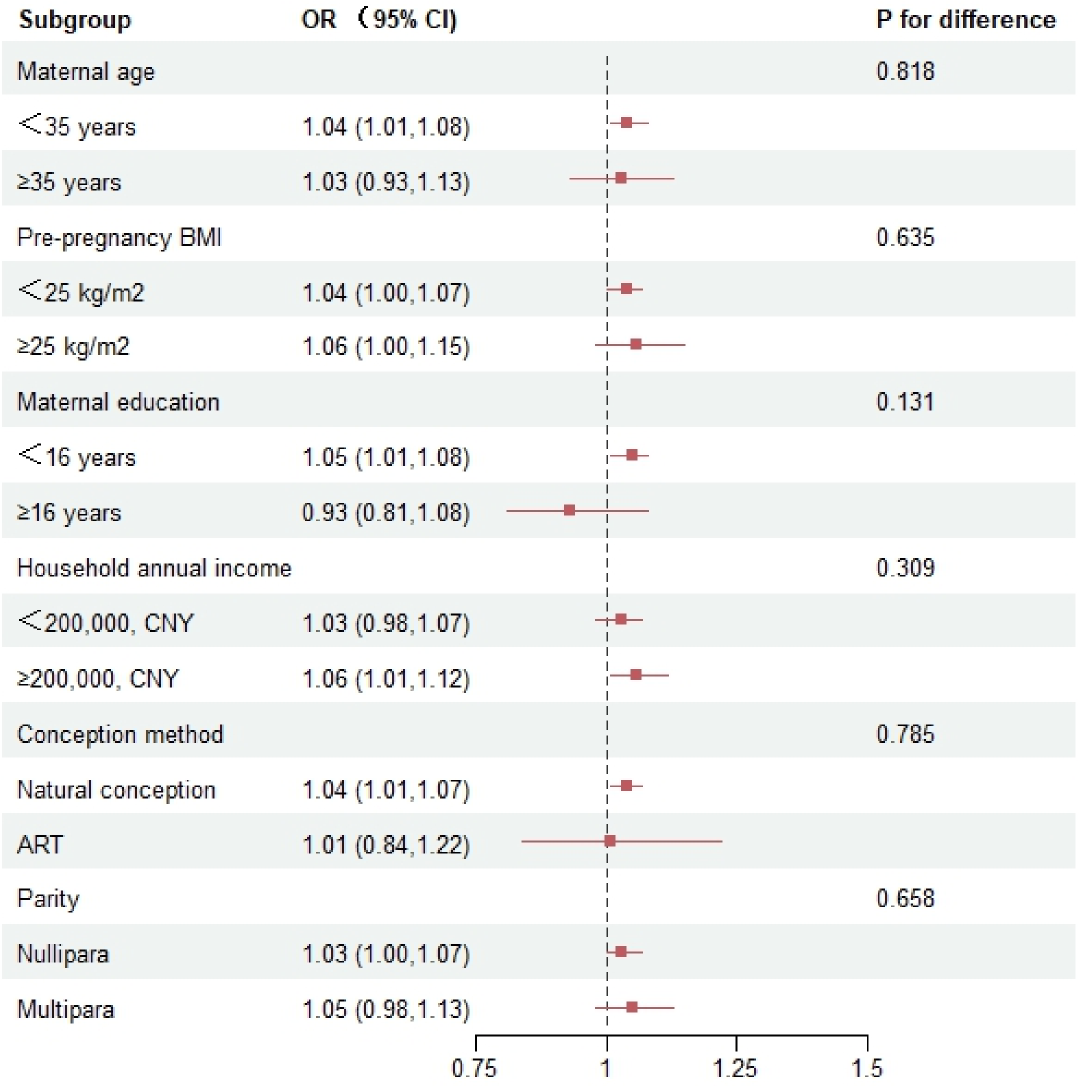

Figure S1 Association between TSH levels and preeclampsia or eclampsia in different subgroups

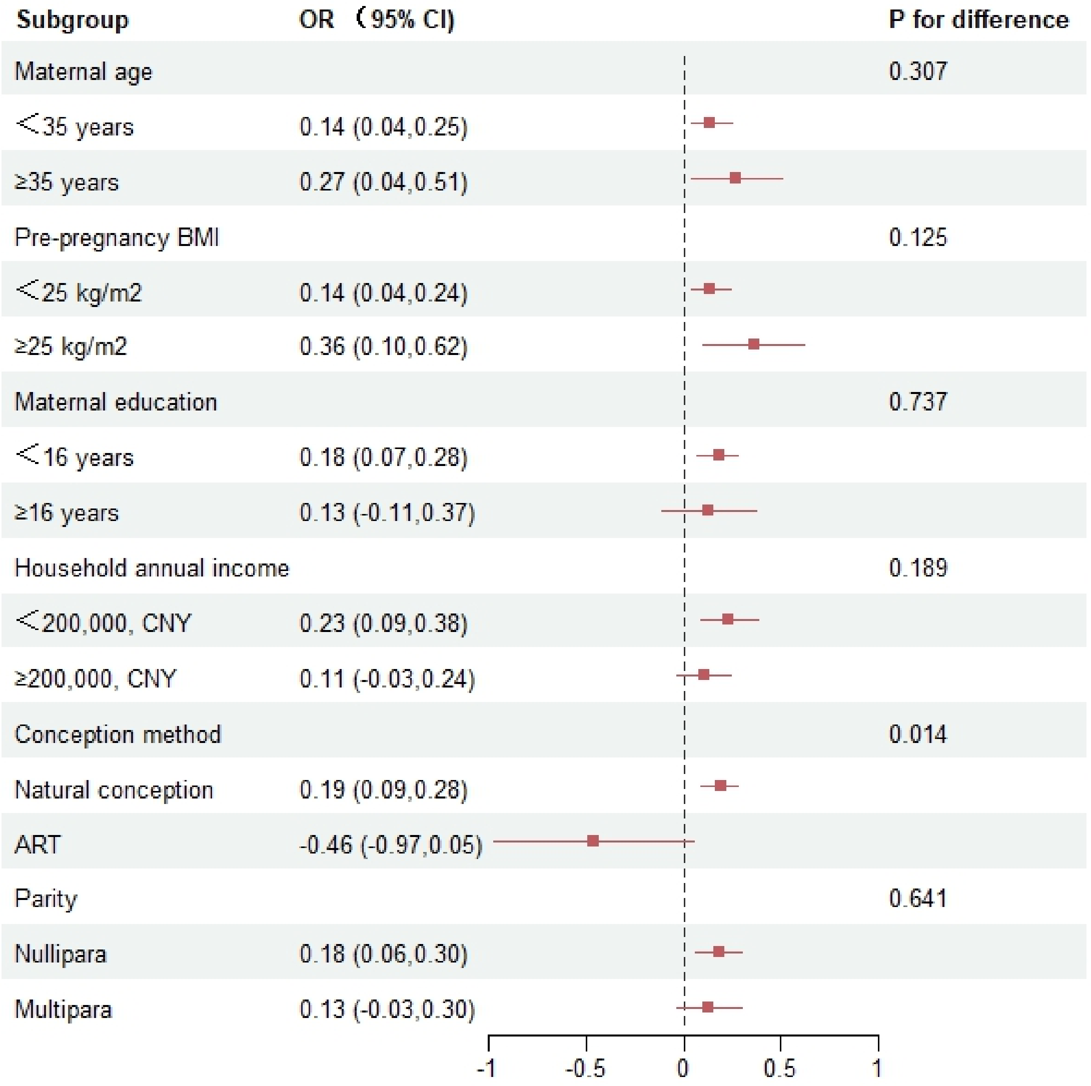

Figure S2 Association between TSH levels and SBP in different subgroups

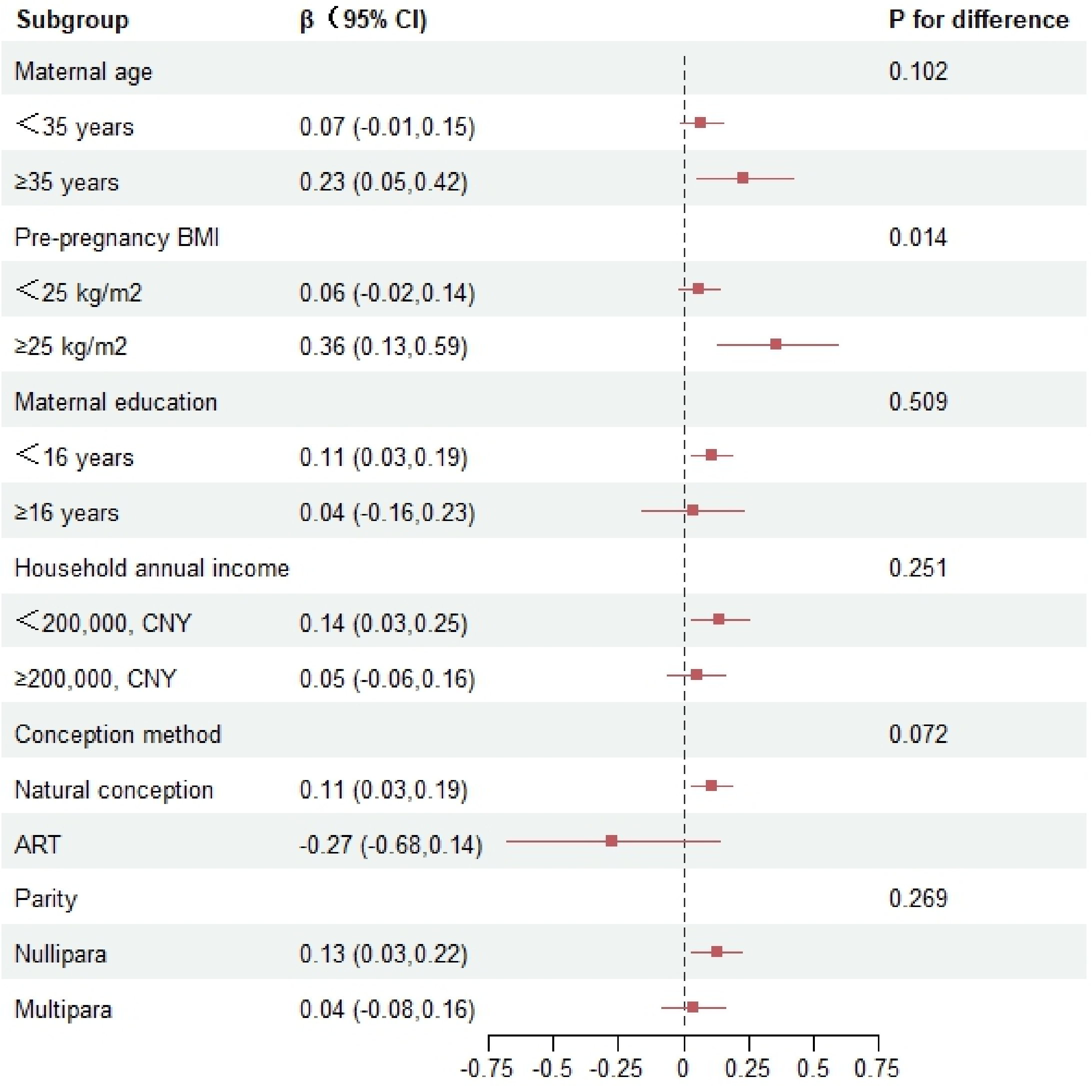

Figure S3 Association between TSH levels and DBP in different subgroups

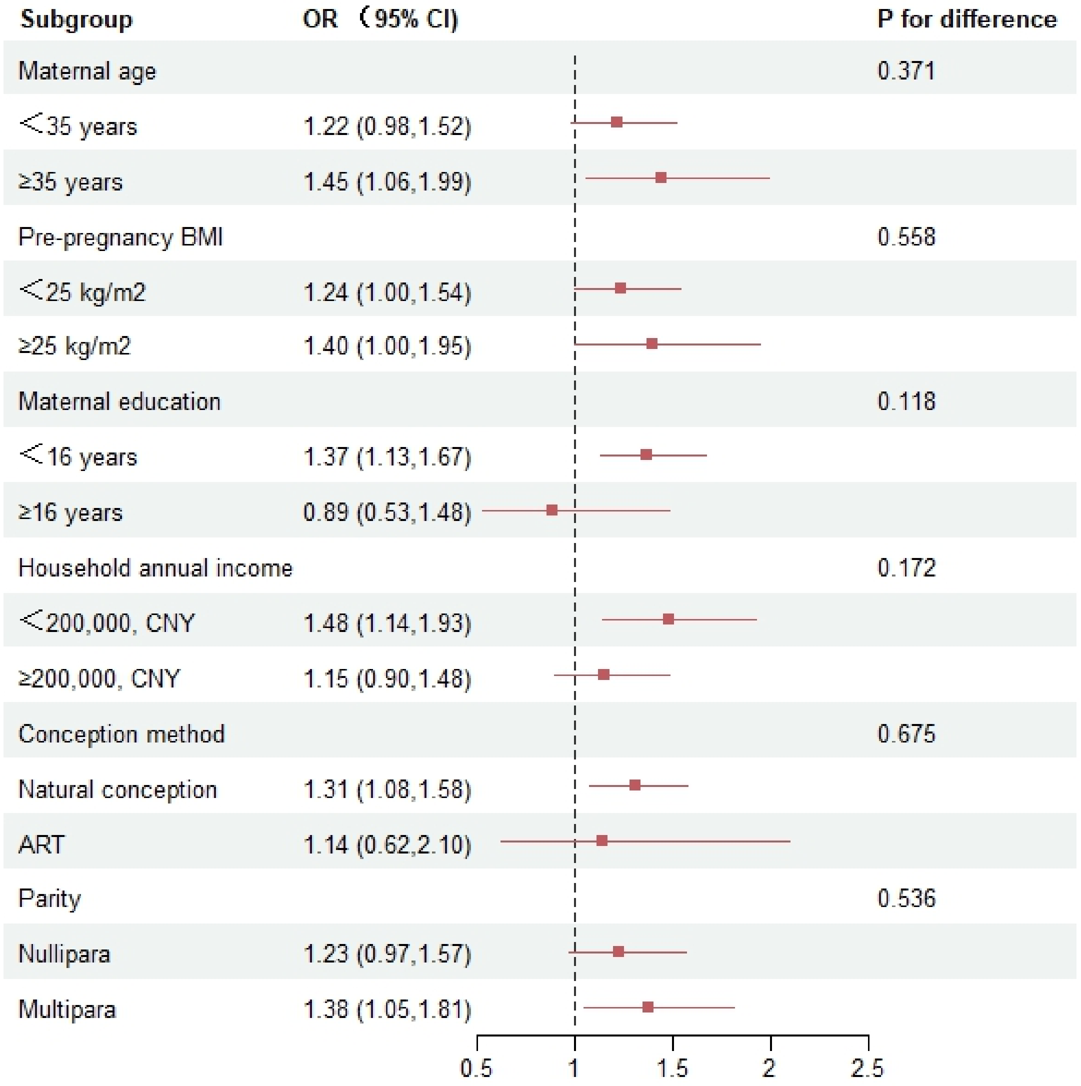

Figure S4 Association between TPOAb positivity and preeclampsia or eclampsia in different subgroups

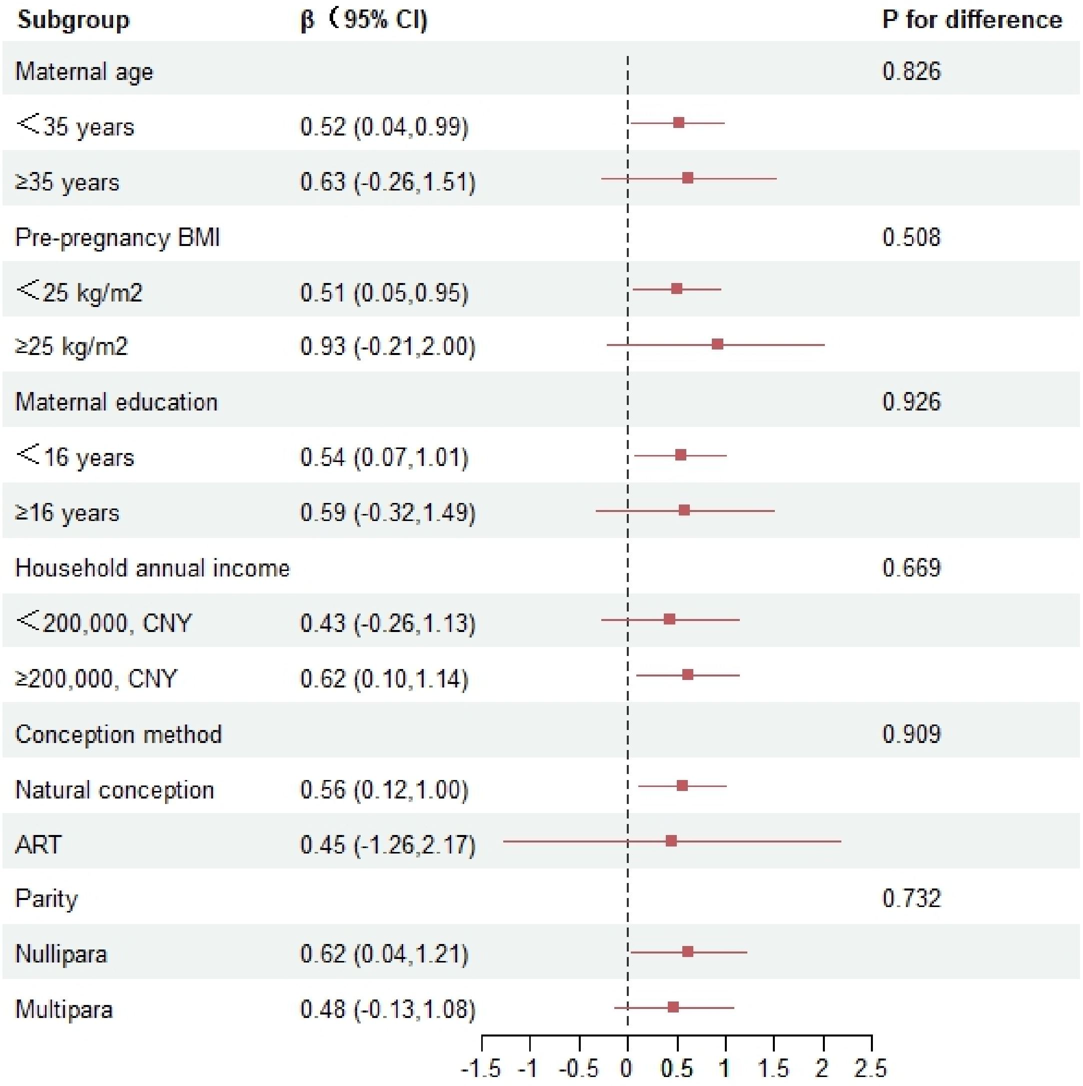

Figure S5 Association between TPOAb positivity and SBP in different subgroups

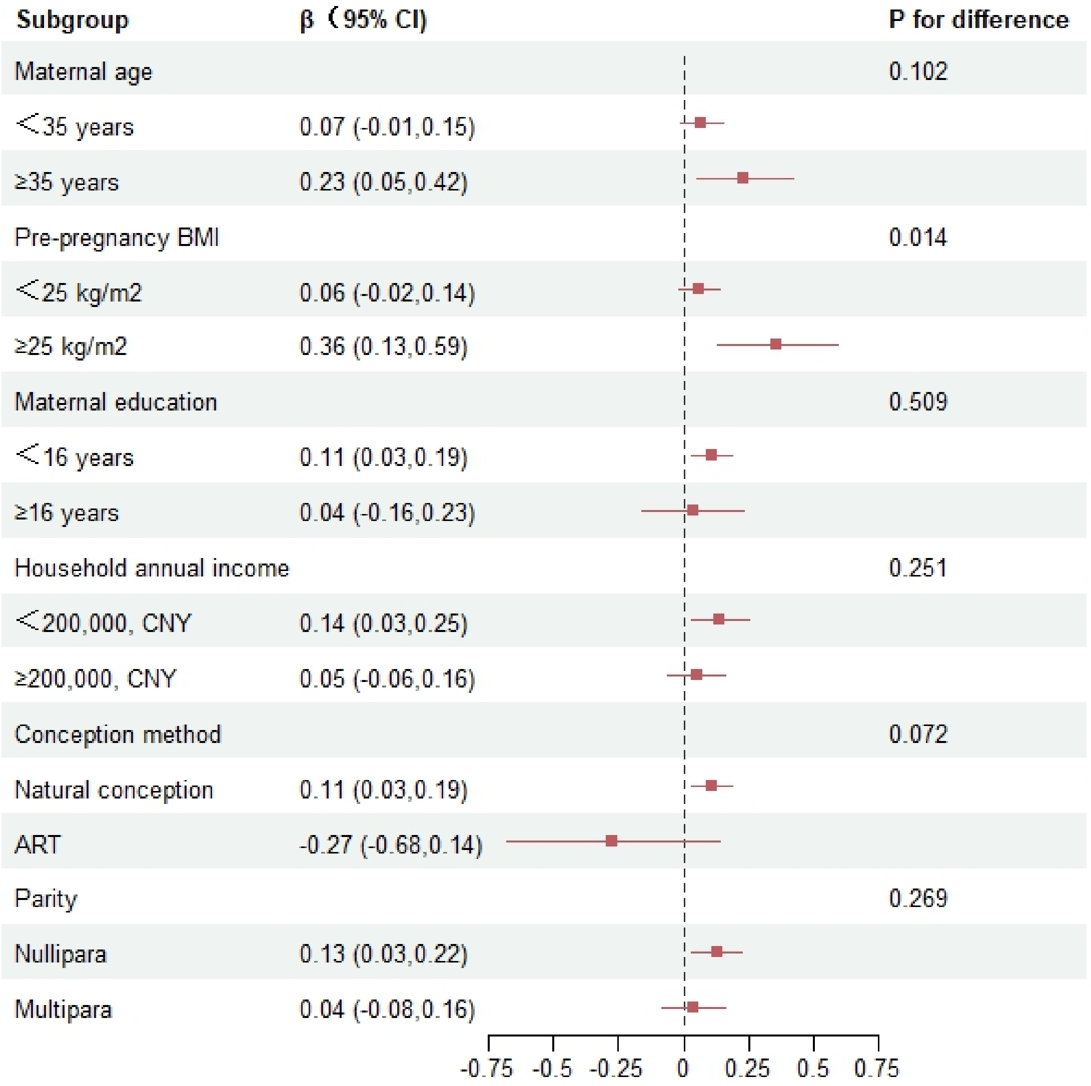

Figure S6 Association between TPOAb positivity and DBP in different subgroups
